# Medical and Physical Intelligence

**Published:** 1823-05

**Authors:** 


					medical and physical intelligence.
MORBID ANATOMY.
1. Case of Perforation of the CEsophagus.?A WOMAN, aged
twenty-two, was admitted at La Pitie, on the 3 1st of January, 1823.
She had been put to bed a few days before at the Maternite, where she
was affected with puerperal peritonitis, for which thirty leeches had
been applied. She was but imperfectly recovered when she left the
Lying-in Hospital; and, after some days, applied at La Pitie under the
following symptoms:?The face pale and anxious; the skin dry;
the pulse small, frequent, and contracted; the abdomen slightly
Medical and Physical Intelligence. 435
distended, and painful. The patient augured very unfavourably of the
result.?February 1st. Thirty leeches were applied to the belly, aud a
simple tisane administered.?2d. The patient had frequent vomiting:
twenty leeches were applied, and an anti-emetic mixture. The vomit-
ing ceased, and she appeared better. At two o'clock, she was agitated
in consequence of a visit from some of her friends, and the receipt of a
letter from her lover: the visitors had scarcely left her, when she
expired.
The body was opened thirty-six hours after death. In the external
appearance there was nothing worthy .of remark, except some distention
of the abdomen, which was filled with a considerable quantity of puru-
lent serosity, with albuminous flocculi. Slight adhesions existed be-
tween the intestines and the abdominal peritoneum. The stomach and
intestines were empty, and white internally. The uterus had still the
size of a large pear, containing some clots of blood, and~~Raving the
vascular orifices still open. On opening the chest, both cavities were
found filled with a dark brown fluid ; and, at the distance of three lines
from the cardia, the posterior part of the oesophagus was perceived to
be completely destroyed, to the extent of four finger's breadth: nothing
remained of this portion but some black sphacelated shreds. The
gangrene pervaded the mucous membrane for two inches further up-
wards, but without involving the other textures. The pneumo-gastric
nerve was seen white and isolated, in the midst of the parts destroyed.
The liquid which had escaped into the thorax contained a worm, of the
genus lunibricus, four inches long. No symptoms had led to the sus-
picion of this extensive destruction of the oesophagus.?(Revue Medi-
cate, Fevrier.)
2. Calcareous Concretions in the Veins.?Several ancient as well as
modern authors have related instances where small stones have been
found in the veins : in addition to the examples already recorded, Dr.
Friedrich Tiedman relates, that he has frequently met with them
in the veins of the bladder, the uterus, the vagina, and the rectum.
They are found in both sexes, especially in persons of a middle and
advanced age; seldom in young people, and never in children. Ac-
cording to the chemical analysis of Professor Gmelin, these stones
consist chiefly of phosphate and carbonate of lime, mixed with animal
matter: they do not contain any uric acid whatever. Dr. T. believes
that these vein-concretions are formed from the blood itself} in the
cavities of the varicose veins. Whether they are likely to excite any
particular morbid effects on the human constitution,?a circumstance
which it would be very useful to know,?has not yet been sufficiently
ascertained.?(Medicinisch-Chirurgische Zciiung.)
3. Calcareous Concretions in the Choroid Plexus.?On raising the
calvarium, and cutting through the dura mater, the vessels of the pia
mater did not appear gorged, but adequately filled with blood; out, on
slicing off the hemispheres, cutting through the centrum ovale, and lully
exposing the ventricles, the consequences of a congested brain were too
evident, in an immense quantity of serous fluid, and a pultaceous
456 Medical and Physical Intelligence.
consistence of the cerebral substance; (emollient to a degree of obscur-
ing the subjects of descriptive anatomy.) But what particularly struck
me as extraordinary, was the existence of two calcareous concretions,
about the size of a pea each, enveloped in the reticulations of the plexus
choroidea, where it hangs over the tenia hippocampi, in the descending
cornu of the lateral ventricle, one on each side: in this situation, the
choroid plexus had the paleness of a maceration in the water of the
ventricle.
Although I saw this case when, alas! the symptoms, too firmly esta.
blished, admitted not of cure; and although the alvine discharges had
the usual appearance of a dark green, as is common in such cases ; yet
it is a question, whether the calculous concretions within the head, or
the disturbance of the organs below the diaphragm, were the causes of
the inflammation of the brain ??(Extract of a letter from Mr. Aspray,
of Olney, December 1822.)
PATHOLOGY.
4. Curious Affection of the Heart.?Dr. Fjsher, of Hildburg-
hausen, relates a case of disease of the heart, in which both the patient
himself and also the by-standers, among whom was a physician, could
distinctly hear the beating of that organ for six or eight weeks previous
to death. They were not only sensible of the noise in the region of the
heart, but, on feeling the pulse at the same time, it was observed that
the pulsations were simultaneous with it. This noise had a great re-
semblance to the croaking of a frog, and it could be heard easily at the
distance of five or six paces from the patient.
On examination after death, the auriculum dextrum and the vena
cava ascendens were found dilated to a considerable size. The right
ventricle contained a firm polypus, of about an inch in breadth ; one
division of which protruded into the auriculum dextrum, the other into
the arteria pulmonalis.?( Medicinisch Chirurgische Zeitung.)
5. Case in which a foreign Body remained seven Years in the
Trachea.?The following case we copy from the Quarterly Journal of
Foreign Medicine for April:
In 1821, a young man, aged seventeen, applied to Walther, af-
fected with phthisis, from the claw of a craw-fish which lie had swal-
lowed in 181J, and which had got into the trachea, and remained in
the right bronchia, occasioning violent convulsions, coughing, and hae-
moptysis. Oil of sweet almonds, followed by opiates, purgatives,
assafcetida, and corrosive sublimate, eased the cough; and lie thought,
as he became easier, that the claw must have been removed. There
remained, however, phthisis, which was followed by cramps, which did
not yield to bathing. Up to 1814, he expectorated pus, and had re-
peated feverish attacks, in spite of pitch-vapour baths and Dover's
powder. lie had subsequently a brain-fever, strong convulsions, cho-
rea, strabismus, and somnambulism. Some time after he was seized
with an irresistible desire to bite, and, when he could find nothing else,
he bit his own hands, lie afterwards had distressing optical illusions,
Medical and Physical Intelligence. 437
and could not look on any thing black without screaming. He lost the
sense of taste, and it was painful for him to utter a sentence. His limbs
were then paralysed; and he took a great passion for cutting and pierc-
ing instruments, and, when alone, he cut and stabbed himself. Some-
times he sought to strangle himself; but, after the excess of the furious
fit was over, he complained of great fatigue, and of uncommon pains. For
two years he amused himself like an infant, during all which time the
cough and expectoration continued. After a time his paralysis was
partly removed again, and he began to walk and to try medicines, all
of which he had given up. Every thing was tried,?blisters, anthel-
mintics, camphor, musk, bark, caiomel, aloes, hellebore, laurel water,
iron, zinc, &c. and all without success. In 1815, his nervous symp-
toms returned during winter, and disappeared again without medicine
in spring. In autumn they again returned, more violently than ever, and
his expectoration contracted a very remarkable fcetor. He bruised
every thing he could come at, and had a strong propensity to leap out
of the window, which he once accomplished from the first floor. His
appetite was gone, and his bowels torpid. In January, 1816, he lost
his voice, his sight, and his hearing; but these he recovered in the suc-
ceeding summer; and, the year following, the same circumstances
occurred. In IS 18, he lost his appetite, and could only take bread,
honey, and coffee. Medicines were again tried, with no avail.
On the 27th of April, ISIS, after a violent coughing for several days,
he brought up the claw of the craw-fish, with a great quantity of pus ;
and this put him in great hopes of a cure. In 1819, he had a quotidian
fever, which was subdued by bark, myrrh, and acetate of lead. In
1S20, he took 110 medicine. During the winter of 1821, he complained
of pains in his side, and spit up blood. He tried the vapour of tar and
sulphur: the expectoration ceased, he recovered his health, and can
now attend to his business.?(Grafe and Walther's Joarn. tier
Chir. und Augenhcil.)
THERAPEUTICS.
0. Croup cured by Sulphate of Copper.?Dr. H. Hofmann re-
commends cuprum sulpliuretum as an excellent remedy in croup,
especially after blood-letting. In slight cases, lie begins with giving
from one quarter to one half a grain every two hours. In those
cases, however, where there is also laryngitis or bronchitis, three, four,
or more grains are administered, so as to excite instant vomiting : by so
doing, the Doctor thinks that not only the lymph is expelled from the
trachea, but also that the further secretion of it is prevented, so that
the patient is very much relieved, and soon cured. After copious vo-
miting has been produced, the medicine is to be given in small doses, in
conjunction with digitalis. In support of the utility of the above prac-
tice, Dr. If. affirms that he has employed it with the greatest success
during a period of ten years, in a great number of children affected
with croup, without ever having lost a single patient in that time; not-
withstanding the disease was often at its height when he was lirst called
FtLAND's Journal.)
1
438 Medical and Physical Intelligence.
7. Treatment of Dysentery.?Professor Wendt, physician to the
General Hospital of Copenhagen, has lately made trial of the Trium-
fetta Semitriloba in dysentery, and reports very favourably of its effects.
It is used in the form of a decoction, which is mucilaginous, without
any degree of astringency or bitterness. It is likewise employed as a
domestic remedy in the West Indies, and is said to prove efficacious
\vhen all other medicines have failed.?(Nye Ilygcea, Januar.)
8. Puerperal Convulsions cured by Ergot.?The Secale Cornutum,
or ergot of rye, has for some years enjoyed considerable reputation
among our American brethren in certain puerperal cases, to which we
shall draw the attention of our readers more particularly on a future
occasion. Mean time we subjoin a case illustrative of its use, related
by Dr. Waterhouse. .
Mrs. L. H., of nervous temperament and uehcate habit, agecl
nineteen, was, on the 24th of June, 1814., seized with the usual pre-
cursory symptoms of parturition. I found her affected with wandering
pains of the back and abdomen, some throbbing pain of the head, and
a tense pulse, though natural in frequency. The loss of fifteen ounces
of blood, with fomentations to the abdomen, and a dose of opium,
gradually gave her relief; and at evening she fell into a quiet and re-
freshing sleep. The next morning I was sent for in haste, and was
informed that, after a quiet night, she discovered in the morning some
symptoms of derangement. She complained of wandering pains in the
abdomen, and of a throbbing sensation in her head. These symptoms
increased, till the most horrid forms of puerperal convulsions were
brought on that I ever witnessed. She was constantly muttering tilings
in the most incoherent manner; her eyes were rolling from side to side,
and turning up in their sockets. She had so frequently bitten her
tongue, that the blood was flowing profusely from her mouth; her ex-
tremities were of a deadly coldness; and the violent spasms and conr
tractions of the muscles of her limbs, back, abdomen, neck, and lower
jaw, were truly alarming. The pulse was natural, but less frequent
than in health. With much difficulty, her lower extremities were im-
mersed in warm water, and large quantities of the tinctures of opium and
assafcetida were forced down. Her abdomen was fomented, and her
extremities smartly embrocated with stimulating applications, &c.; but
all to no purpose. There was no hemorrhage; but, from the condition
of my patient, it was impossible to make that accurate examination per
vaginam that I wished. I could, however, ascertain that the os uteri
was in a small degree dilated. The circumstances were so urgent, that
I could not defer the use of means till 1 could procure a consultation.
Her strength was rapidly wasting, pulse small and frequent, breathing
laborious, and countenance ghastly. The ergot presented itself to my
mind as the only probable means of saving her life. I mixed thirty
grains in a small quantity of warm water, and gradually insinuated a
table-spoonful between her teeth, worked it into her mouth, and in two
or three minutes she had swallowed it. The effects were almost instan-
taneous, and truly astonishing. Her spasms gave way, the operations
of her mind became regular, and she awoke, as she supposed, from a
Medical and Physical Intelligence. 439
disturbed and painful sleep. She complained of much weariness. A
strong cup of tea was given her, with some light nourishment, and she
soon fell into a quiet sleep. In the evening following, true and forcible
labour-pains came on, and I delivered her in a short time with perfect
safety.?(Philadelphia Journal, No.ix.)
9. Case of Poisoning with Arsenic.?The following case tends, in
some degree, to confirm the opinion of Mr. Hume, as given to the
public in various Numbers of this Journal:
" On the 8th 01 December last, (says Mr. Buchanan^) I was re-
quested by a woman to see her husband immediately: she feared he
had taken poison, as he had vomited some white powder, and was iu
much pain. I found him rolling on the floor, and, by his actions, ex-
pressing great pain at his stomach and nausea. The matter he had
vomited seemed to be chiefly water, but having a considerable deposit,
consisting of perhaps two or three drachms of powder. He could give
no account of it, or of himself. His pulse was languid ; his tcngue and
mouth clean ; his skin was covered with cool perspiration. In the
course of fifteen or twenty minutes, I gave him, in divided portions, a
solution of four drachms of sulphate of zinc in six ounces of water.
As this was much against his will, some of it was lost; but three drachms
of the salt were taken before free vomiting took place, when two or
three more drachms of the powder were brought up. Until this time
110 other fluid had been given: it was not easy to get him to take
what was judged the best remedy, the emetic; and it was thought the
giving of fluids might increase the activity of the poison. Now that
vomiting was frequent, he was allowed warm water, and no more of
the white powder was present when it was rejected. He took some
cathartic pills, and half-drachm doses of pure magnesia,?a course of
practice recommended by Mr. Hume, with whom it had been success-
iiil. I was not aware ol its manner of operation, except it might render
the arsenic, or white lead, less soluble; and one of these substances,
from its gravity, (the only means I then had of estimating it,) I judged
it to be. On examining the powder, of which I still possess a quantity,
I had no reason to doubt it was the white oxyd of arsenic. I sublimed
some; I formed Scheie's green with copper by another part, and I was
satisfied of its garlicky odour on being heated. My patient took seven
or eight half-drachm doses of the magnesia: his vomiting ceased soon
after its first exhibition ; his bowels were soon well cleared; all pain
rapidly subsided; and on the next day he had no other complaints than
might be fairly attributed to the soreness produced by vomiting, and the
distress resulting from his conduct.
" Since so much of the case was penned, I have had an opportunity
of ascertaining that he obtained at a druggist's in the neighbourhood an
ounce of arsenic : lie mixed it with milk and water, and swallowed very
nearly the whole. In about half an hour vomiting began, and a few
minutes after that period I saw him. It is assuredly a curious cir-
cumstance that so large a portion of so violent a poison, (and its
genuineness has been ascertained by a reference to the druggist who
dispensed it, and by the examination of Dr. Babington,) should have
440 Medical and Physical Intelligence.
been taken into the stomach, and not have produced more serious
effects. It is not for me to say what share in preventing it should be
attributed to the full, free, and almost dry, vomiting induced ; or what
is the probable value of magnesia as an antidote; my object, in giving
this recital, having been merely a statement of the facts.?(Medical
Repository, April.)
SURGERY.
10. New Method of performing the Operation of Lithotomy in the
Female, by Dr. Lisfranc.?The patient, in this operation, is placed
in the usual position, and a common catheter is passed into the blad-
der. Its convexity is then turned upwards; the handle is given to an
assistant, who, pressing lightly from above downwards, depresses the
urethra and vagina. The part to be operated upon is that space lying
between the urethra and the symphysis of the pubis. The surgeon,
having ascertained the position of the two rami of the os pubis and of
the clitoris, passes his finger into the vagina, in order to ascertain the
course of the pudic artery, which is occasionally irregular. The ope-
rator then taking a common bistoury, and holding it like a writing-pen,
makes a semilunar incision, (the convexity being upwards,) marking
with his left hand the spot where the incision should begin and end.
It is to begin on a level with the right side of the meatus urinarius, one
line distant from that branch of the pubis, and is carricd to the same
point on the other side. It is recommended to cut the cellular tissue
layer by layer. It is extremely important not to make such a degree
of pressure on the anterior surface of the bladder as to detach it Iroin
the pubis. The surgeon having reached the anterior and inferior part
of the bladder, may open it transversely, after having passed his bistoury
into the cavity; but, if the thumb and the fore.linger of the left hand
are introduced, (the first in the vagina, and the second into the wound,)
and the parts between are a little stretched, the bladder will become
tense, he draws a little forwards, and the transverse incision will be
more readily and easily made. Repeated experience, it is said, has
proved that there is no danger, in following this method, of wounding
either the urethra or the pudic arteries; and it is impossible to injure
the vagina. It is to be observed, that there is less danger of hemorrhage
from a wound in the body of the bladder, than in its neck. The wound
made by this operation is capable of greater extension than that made
in the ordinary manner, and, consequently, is more advantageous for
extracting large calculi. The infiltration of the urine appears to be
impossible, for the following reasons: 1st, because the bladder is
situated higher in the woman than in man; 2dly, because the cellular
membrane between the bladder and pubis is fine, elastic, and in small
quantity; 3dly, because the shortness, breadth, and position, of the
urethra favour the escape of the urine by the natural passage. Inflam-
mation of the bladder or peritoneum are not, of course, more to be
feared in this than in the common mode of performing the operation.-?
(Journal Universe!Fevrier 1823.)
Medical and Physical Intelligence. 441'
11. A new Instrument for the Operation of the Cataract, invented
by Dr. Bancal, (Kystitome Cache.)?This instrument is an improve-
ment upon the kystitome of Lafaye, the inconveniences of which
were numerous, arising from the roundness of the handle, the want of
the means of apportioning the length of the blade, which should be
pushed out from the sheath, and the force which was necessary for
effecting this object, and which caused a vacillation, highly desirable to
be avoided in the midst of such delicate parts. Dr. Bancal, who had
performed numerous operations with this instrument, at the Isle of
Bourbon, at Calcutta, and in other parts of India, was so struck with
these imperfections, that heat length invented the instrument which we
are now about to describe, and the mechanism of which is entirely his
own contrivance. It is composed of a flat, long sheath, having a little
projection at its upper extremity, from which a small cutting blade is
made to issue .by pressing on a button placed at the side, and which is
rendered inoffensive by a little tongue attached to the upper part of the
sheath. The instrument is held like a pen; it is introduced in the same
manner as the kysist6me of Lafaye, without any risk to the parts over
which it has to pass; and it requires no effort or violence to cut that
part of the membrane of the chrystalline which the operator wishes to
divide. Whenever it is necessary to detach the chrystalline lens from
its attachments, to divide or to open the thickened capsule, or to destroy
the adhesions which it has contracted, no instrument can be superior to
that of M. Bancal; of which, some trials lately made at Paris, confirming
the satisfactory results previously obtained at Bourdeaux, will no doubt
very much extend the reputation and use. M. Bancal thinks this in-
strument may be very advantageously employed in the operation for
artificial pupil; and we are well disposed to believe this to be the case,
from the explanation he has given in his memoir.*?(Journal Univer-
sel, Fevrier.)
12. Case of Trepan.?On the 1st of February last, M. Dupuytren
performed the operation of trepan upon a patient in the Hotel Dieu at
Paris, under very peculiar circumstances. The patient, a young man,
had two years before received a stroke with a pointed knife, over the
upper and back part of the right parietal bone. For this wound he was
admitted into the Hotel Dieu: it soon healed. The patient, for a long
time after, experienced no bad symptoms; when at length a tumor ap-
peared upon the spot where the blow had been inflicted. This tumor
suppurated and burst, and a piece of the point of the knife, an inch
and a half in length, was discharged through the wound, two months
and a half before the date of the operation. M. Dupuytren was of
opinion that the knife must have penetrated through the bone into the
substance of the brain. The discharge of pus soon ceased; the opening
closed entirely; but the patient began to complain of pain in the head,
to experience from time to time rigors, and irregular feverish fits; for
which leeches behind the ears, laxatives, pediluvia, &c. were prescribed.
* We despair of giving our readers a very corroct idea of this instrument,
without the assistance of a plate.?Editors.
NO. 291. 3 M
X.
442 Medical and Physical Intelligence.
These symptoms continued to increase in severity; the patient was
struck with total palsy of the left side, and with occasional convulsion
of the muscles of the right: in fine, he lost the powers of speech,
sensation, and intelligence, and lay in a state of profound stupor, with
low stertorous breathing, and with a slow (and what M. Dupuytren
considered a feeble) pulse. Taking all the circumstances into account,
there could be no doubt that the symptoms depended upon compression
of the brain; and, as M. Dupuytren believed, from a collection of pus
between the dura mater and the cranium. The trephine, with the ex-
pectation of finding matter here, was accordingly applied in the situa-
tion of the wound, and a circular portion of bone removed; and the
surprise of the surgeon may be conceived, when the dura inater was
considered to be quite healthy. Observing, however, that it projected
into the wound, M, Dupuytren cut through the dura mater; but he
observed nothing unnatural in the state of the brain below. A bistoury
was then plunged, to the depth of more than an inch, into the substance
of the brain itself, and by this opening an ounce and a half of pus was
discharged. The patient, who had not complained during the incisions
of the scalp, now complained loudly of pain in the wounded parts : he
spoke distinctly, and answered with accuracy the questions put to him;
and the paralysis was so much relieved, that he moved his limbs at
will, and stretched out the paralytic arm to M.Dupuytren, when he
was leaving him. ?For more than forty-eight hours after the operation,
the patient continued to do well, no symptoms appearing either of in-
flammation or compression of the brain. All of a sudden, however, he
fell into violent convulsions, which ended in death in about two hours.
The brain was inspected the second day after, and no marks of inflam-
mation of the membranes, or of the brain itself, were discovered. There
was a small quantity of pus in the cavity of the abscesses, but not enough
to produce any degree of compression.? (Quarterly Journal of Foreign
Medicine, April.)
MIDWIFERY.
13. Case of Super-foetation, by M. Percy.?A woman of Torrigeny,
in the department of the Seine et Marne, became pregnant in 1820, for
the third time. The commencement of the pregnancy presented nothing
remarkable, but about the fourth month she felt the movements of the
child very distinctly, particularly 011 the right side: these, although
at first very strongly marked, by degrees became weaker, and finally
ceased altogether, without any obvious cause. At the end of seven
weeks, she experienced again all the symptoms of a new impregnation,
which excited great anxiety; but the nine months of gestation were
completed without any considerable inconvenience. On the accession
of labour, the pains succeeded each other so briskly that at the end of
an hour a male child was born : it was small, but lively, and afterwards
throve well. Soon after the expulsion of the foetus, which was accom-
plished without any difficulty, fresh pains came on ; during which several
dark, inorganic, coagulated masses were expelled : these were followed
Medical and Physical Intelligence. 44 3
by a black, flocculent, spongy mass, in the centre of which was found a
female foetus at the fourth month, in good condition, and which has
beeu preserved.?(Revue Medicale, Fevrier.)
CHEMISTRY AND PHARMACY.
14. Cinnabar.?M. Kirchoff prepares cinnabar in the following
manner:?Triturate in a porcelain cup, with a glass pestle, 300 parts of
mercury with 68 of sulphur, moistened with some drops of a solution
of potash, till a black proto-sulphuret is formed; and then add 160
parts of potash, dissolved in an equal quantity of water. Heat the
vessel containing the mixture over the flame of a candle or lamp, conti-
nuing the trituration without intermission. Add pure water from time
to time as the liquid evaporates, that the substance may be constantly
covered an inch deep. After two hours' continued trituration, a great
part of the liquid being allowed to evaporate, the mixture begins to
change from black to brown, and then quickly to red. No more water
is to be added, but the trituration is to be continued. The mass will
acquire the consistence of a jelly, and the red becomes more and more
brilliant with great rapidity. YVhen it has attained its highest perfec-
tion, the cup should instantly be removed from the flame, or the red
will quickly change to a dirty brown colour.?(Phil. Mag.)
15. Condensation of the Gases.?Mr. Faraday has succeeded in
condensing chlorine into a liquid. For this purpose a portion of the
solid and dried hydrate of chlorine is put into a small bent tube, and
hermetically sealed : it is then heated to about 100, and a yellow vapour
is formed, which condenses into a deep-yellow liquid heavier than water,
(sp. gr. probably about 1.3,) Upon relieving the pressure by breaking
the tube, the condensed chlorine instantly assumes its usual state of gas
or vapour.
When perfectly dry, chlorine is condensed into a tube by means of a
syringe; a portion of it assumes the liquid form under a pressure equal
to that of lour or five atmospheres.
By putting some muriate of ammonia and sulphuric acid into the op-
posite ends of a bent glass tube, sealing it hermetically, and then
suffering the acid to run upon the salt, muriatic acid is generated under
such pressure as causes it to assume the liquid form: it is of an orange-
colour, lighter than sulphuric acid, and instantly assumes the gaseous
state when the pressure is removed.
By pursuing this mode of experimenting, sulphuretted hydrogen,
sulphurous acid, carbonic acid, cyanogen, euchlorine, and nitrous
oxide, have been also found to assume the liquid form under pressure,
and to appear as limpid and highly mobile fluids. It is probable that
other gases may be condensed by similar means, and that nitrogen,
oxygen, and even hydrogen itself, may yield, provided sufficient pres-
sure can be commanded. Some of Mr. Perkins's experiments render
it more than probable that atmospheric air, under a pressure ot some
hundred atmospheres, changes its form ; and it is not unlikely that
some very curious and interesting results may be obtained by the aid of
444 Medical and Physical Intelligence.
a slight modification of the apparatus used by that gentleman, in his re-
searches connected with high-pressure steam.?(Journal of Science7
No. xxix.)
16. Dobereiner's Apparatus for making Extracts.?This appa-
ratus serves to extract, by means of water, alcohol, or ether, the soluble
substances from any substance to be analyzed, in quantities from ten
up to 200 grains. It is composed of a tube of glass, from four to nine
lines in diameter, and from four to nine inches long. The tube is closed
below by a cork, to which is adapted a small tube open at both ends.
This, except that its upper extremity is covered with a piece of inuslin,
communicates with the large tube. The substance to be operated upon
is put into the large tube, about half filling it, and the solvent is then
put in over it. A small glass bulb, proportionate in size to the quantity
of solvent used, is then emptied of air by heating a few drops of alcohol
in it, and immediately attached by a tight cork to the lower end of the
small tube. The whole apparatus is then set aside in a cool place: as
the alcohol vapour condenses, a vacuum is produced, and the pressure
of the air in the large tube forces the fluid through the substance to be
operated upon into the bulb. In a few minutes the extraction is com-
plete ; the bulb is then removed, its contents taken out, the air in it
again displaced, and the operation repeated : or, if necessary, the fluid
is left in contact with the substance some time before it is made to pass
from it into the bulb. ? (J our. of Science, from the Bib. Univ. xxi. 188.)
17. English Opium.?Messrs. Cowley and Staines, of Winslow,
Bucks, have cultivated poppies for opium with such success, as to in-
duce the belief that that branch of agriculture is of national importance
and worthy of support. In the year 1821, they produced sixty pounds
of solid opium, equal to the best Turkey opium, from rather less than
four acres and a half of ground. The seed was sowh in February^
came up in March, and, after proper hoeing, setting out, &c. the opium
gathering commenced at the latter end of July. The criterion for ga-
thering the opium was when the poppies, having lost their petals, were
covered with a bluish-white bloom. The scarificator, an instrument
containing five small blades, was then applied to them ; horizontal in-
cisions being preferred, because the juice was not so apt to run from
them before inspissation. After being scarified in one aspect, the head
was left until the juice was coagulated, (about two hours;) it was then
removed by gatherers, and fresh incisions made on other parts. The
poppies were found to produce opium freely until the third or fourth
incision, and some of them even to the tenth. Opium was gathered
daily, until, at the rate of 30s. per pound, the produce would no longer
bear the expence. Ninety-seven pounds and one ounce were procured
at an expence of 3lZ. i is. 2|rf.; and this, when evaporated sufficiently
in the sun, produced above sixty pounds of properly dried opium.
The poppies stood on the stalks until they began to turn yellow (Aug.
18;) they were then pulled, and laid in rows on the land; and, when
sufficiently dry, the heads were gathered, thrashed, and the seed sepa-
rated by coarsc riddles, and cleaned by fine sieves and a fan. The seed
Medical and Physical Intelligence, 445
amounted to thirteen hundred-weight, and was expected to produce
seventy-one and a half gallons of oil. The oil-cake was given to pigs
with great advantage, and also to stall-feeding cattle. An extract may
also be made, by cold infusion, from the capsule of the poppy, eight
grains of which are equal to one of opium: an acre produces eighty
pounds of it. The poppy-straw, when well trodden in the yard, and
laid in a compact head to ferment, makes excellent manure.
The quantity of opium consumed in this country is supposed to
amount annually to about 50,000 ft., exclusive of exportation. This
quantity (say Messrs. Cowley and Staines,) our experiments have con-
vinced us could be easily raised in many parts of Great Britain, where
good dry land and a superfluous population exist together. On the
moderate calculation of ten pounds per acre, that quantity would only
require four or five thousand acres of land, and from forty to fifty
thousand people. The employment would be given to such persons as
are not calculated for common agricultural labour, and at a time when
labour is wanted, namely, between hay-time and harvest.?(Transact.
Soc. Arts, xl. 9.)
MISCELLANEOUS.
18.?Experiments relative to Yellow Fever, performed by M. GuyoN,
at Fort-Royal, Martinique.
1. June 18th, 1822, lie took the shirt of a soldier affected with
yellow fever, which was completely soaked (toute imbibee) in the sweat
of the patient, put it on immediately, and wore it for twenty-four hours.
At the same time he was inoculated in both arms, by M. Cuppe, surgeon
of marines, with the yellow matter, from blisters in a state of sup-
puration.
2. June 30th, M. Guyon drank a small glass holding about two
ounces of the black vomit; and afterwards, having rubbed both arms
with the same matter, was inoculated with it by M. Cuppe.
3. July 1st. A patient having died of yellow fever on the fifth day
of the disease, M. Guyon put on his shirt, impregnated with black
matter still warm, and immediately went into the bed of the deceased,
which was soiled with various excrements. He remained six hours and
a half, sweated, and slept in it, in presence of many witnesses.
4. July 2. The patient who had afforded the opportunity of mak-
ing the first experiment having died, his body was opened. The stomach
contained a pretty large quantity of black matter, of a bloody appear-
ance; and the internal membrane was red and inflamed. M. Guyon
was again inoculated in both arms with this matter, and the punctures
were covered with portions of the diseased stomach. The applications
were removed twenty-four hours after: the inoculated parts were in-
flamed and painful, and the axillary glands somewhat tumefied.
M. Guyon enjoyed uninterrupted health during the performance of
these experiments, which took place before numerous witnesses, and the
authenticity of which is guaranteed by the signature of M. Donzelot,
lieutenant, general and governor.?(Revue Medicate, Fevrier.)
2
446 Obituary.?Mr. Charlton.
19. Remarkable Fact relative to the non-Contagion of the Yelloiv
Fever.?A young Englishman, who arrived at St. Thomas's the preced-
ing year, with a young and beautiful countrywoman whom he had se-
cretly married, was attacked by the yellow fever. When the disease
was at its height, and the symptoms of inevitable death became apparent,
the young woman, in despair, and determined not to survive the object
of her affections, undressed herself entirely, and placed herself by the
side of her dying husband in bed, embracing his body. She remained
for ten hours in this situation, and was with difficulty removed after he
had breathed his last. She did not experience the slightest symptoms
of the disease.?(Revue Encyclopediaue, Jan.)
20. Society for the Relief of Widows and Orphans of Medical Men,
On Saturday, the 5th of April, the Society for the Relief of Widows
and Orphans of Medical Men in London and its vicinity, held its annual
meeting, Dr. Baillie in the chair. The meeting was numerously at-
tended, and we recognized, with great satisfaction, some of the most
distinguished members of the profession, giving their countenance and
assistance to a society which has so strong a claim upon the attention
and patronage of every branch of the healing art, and which, notwith-
standing, has not hitherto received all the support and encouragement
that might have been expected, considering the noble objects, and, we
may say, the excellent management, of the institution. This last meet-
ing was however, in all respects, a most auspicious one. His Majesty,
with a liberality worthy of a monarch, sent one hundred guineas in fur-
therance of the objects of the charity: other handsome donations were
also made; and twenty-three new members were added to the society,
the greatest number that had ever been named on any one day.?We
trust that this short paragraph may not have been written in vain, but
that those gentlemen who are not yet acquainted with the nature and
objects of this society may be induced to institute those inquiries con-
cerning it, which cannot fail to terminate in their enlisting themselves
under its banners.
METEOROLOGICAL JOURNAL,
From March 20, to April 19, 1823.
By Messrs. William Harris and Co. 50, Holborn, London.
Rain
gauge
Mar-
20
21
22
23
24
25
26
27
28
29
30
31
Apr.
1
2
6
7
8
9
10
11
12
13
14
15
16
17
18
19
.38
.11
.89
.95
29.58
29.22
28.93
29.31
30.09
30.08
29.93
30.00
29.80
29.85
29.80
29.93
29.90
29.75
29.61
29.03
28.90
29.13
29.52
29.68
29.69
29.88
30.01
30.00
29.93
29.92
30.10
29.91
29.74
29.47
29.40
29.32
29.13
29.14
29.75
30.13
30.02
29.88
29-80
29.83
29.81
29-74
29.95
29.81
29.53
29.50
28.96
28.92
29.38
29.62
29.64
29.75
29.87
30.08
29.98
29.90
30.01
30.13
29.85
29.67
29.31
29.47
UG
LUC'S
HYG.
9 A.M.
SYVvar
S W
sw
WNW
NN W
E
NE
E
ESE
NNE
SE
W
WNW
WSW
SW
S
W
w
NNE
NE
NE
ENE
E
SEvar.
ENE
E var.
SWva.
SW
SW
WNW
NNW
10P.M.
WSW
SW
WSW
NNE
NNE
E
ENE
SSE
E
E
WSW
WSW
W SW
WSW
SW
ssw
WSW
NNW
E
NE
ENE
ESE
E
E var.
SE
E var.
WSW
WSW
W var.
W var.
NW
ATMOSPHERIC
VARIATION.
9 A.M.
Snow
Rain
Overc.
Fair
Fine
Foggy
Foggy
Fail-
Foggy
Overc.
Fail-
Fine
Fine
Fine
Fine
Rain
Sho'ry
Overc.
Rain
Fair
Cloud.
Fine
Fine
Fine
Fine
Fail-
Fair
Faii-
Fine
V. fine
Fair
2 P.M.
Rain
Slio'ry
Fair
Fine
Fair
Faii-
Fine
Fine
Fair
Fine
Sliow'r
Rain
Cloud.
Fine
Fine
Fine
H.&R
Ha.Sh
The quantity of rain during the month of March,
was 1 inch and 16.100ths.

				

